# An Efficient Neuro-framework for Brain Tumor Classification Using a CNN-based Self-supervised Learning Approach with Genetic Optimizations

**DOI:** 10.2174/011570159X378103250806214613

**Published:** 2025-09-18

**Authors:** Paripelli Ravali, Pundru Chandra Shaker Reddy, Pappula Praveen

**Affiliations:** 1 School of Computer Science and Artificial Intelligence, SR University, Warangal, Telangana, India;; 2 Department of CSE, Amity School of Engineering and Technology, Amity University, Noida, Uttar Pradesh, India

**Keywords:** Glioma classification, self-supervised learning, MRI-Imaging, deep learning, genetic optimizations, neurobiology

## Abstract

**Introduction:**

Accurate and non-invasive grading of glioma brain tumors from MRI scans is challenging due to limited labeled data and the complexity of clinical evaluation. This study aims to develop a robust and efficient deep learning framework for improved glioma classification using MRI images.

**Methods:**

A multi-stage framework is proposed, starting with SimCLR-based self-supervised learning for representation learning without labels, followed by Deep Embedded Clustering to extract and group features effectively. EfficientNet-B7 is used for initial classification due to its parameter efficiency. A weighted ensemble of EfficientNet-B7, ResNet-50, and DenseNet-121 is employed for the final classification. Hyperparameters are fine-tuned using a Differential Evolution-optimized Genetic Algorithm to enhance accuracy and training efficiency.

**Results:**

EfficientNet-B7 achieved approximately 88-90% classification accuracy. The weighted ensemble improved this to approximately 93%. Genetic optimization further enhanced accuracy by 3-5% and reduced training time by 15%.

**Discussion:**

The framework overcomes data scarcity and limited feature extraction issues in traditional CNNs. The combination of self-supervised learning, clustering, ensemble modeling, and evolutionary optimization provides improved performance and robustness, though it requires significant computational resources and further clinical validation.

**Conclusion:**

The proposed framework offers an accurate and scalable solution for glioma classification from MRI images. It supports faster, more reliable clinical decision-making and holds promise for real-world diagnostic applications.

## 1 INTRODUCTION

Gliomas are one of the most common aggressive intracranial neoplasms. Thus, precise grading is essential for effective treatment strategy decisions and a good prognosis for patient outcomes. The precise grading of gliomas with non-invasive MRI methods would considerably ease early diagnosis and, thus, the assessment of prognosis with therapy planning in time. This, however constitutes very challenging work to accomplish since the morphological nature of the gliomas comprises complex alterations that call for advanced and sophisticated methods for image analysis [1]. These have been shown to work well in the feature extraction and classification by many conventional supervised deep learning architectures. However, it requires large amounts of highly labeled datasets [2]. The opposite often applies in the domain of medical imaging application which are usually scarce in well annotated data is adequately, because acquiring them involves significant and sometimes prohibitive conditions; they require a significant number of human experts due to the complexity of the involved processes, and developing a model that is both generalized and highly accurate presents major challenges [3].

A current series of works has adopted the use of pre-trained CNNs such as VGG-16, ResNet-50, and Inceptionv3 to classify tumorous regions with acceptable accuracies. However, these models typically are not as strong or computationally efficient in many high-stakes applications involving clinics. Traditional supervised methods in [4] tend to overfit to small data sizes, leading to poor performance on test samples. Current advancements with self-supervised learning and transfer learning offer promising avenues to overcome current limitations. With approaches such as SimCLR, frameworks that apply contrastive learning have potential in extracting meaningful representations from unlabeled sets thus diminishing reliance on annotation sets. Such learned techniques, coupled with effective clustering and ensemble methods can form an efficacious solution for classifying gliomas [5]. In this context, it suggests the following comprehensive efficient workflow with self-supervised feature extraction, clustering approach, transfer learning, ensemble classification, and evolutionarily optimized parameters.

Specifically, the SimCLR is a self-supervised contrastive learning framework used for pretraining MRI images in order to enhance feature extraction from unlabeled data samples. These features are subsequently clustered with Deep Embedded Clustering, producing structured representations aligned with the grade of gliomas. The clustered data are then further processed within EfficientNet-B7, which is a highly parameter-efficient CNN architecture and thus produces intermediate classifications [6]. Lastly, an ensemble of EfficientNet-B7, ResNet-50, and DenseNet-121 leverages the strength of each one but Differential Evolution (DE) optimizes the parameters of the Genetic Algorithm (GA) that define the ensemble hyperparameters. A study like this can, therefore, be considered a valuable framework for providing a noninvasive process in glioma grading with high computational accuracy [7].

It was mainly motivated by the fact that existing glioma classification models are usually constrained by limited labeled data, low computational efficiency, and poor generalization. Most CNN-based models for MRI image classification require significant amounts of labeled data, which are expensive and time-consuming to obtain [8]. Furthermore, they rely on single-model architectures which lack the ability to effectively extract subtle morphological features of gliomas and consequently lack uniform performance over different datasets. Self-supervised learning appears to be a promising alternative with the potential to allow feature extraction in a completely unsupervised scenario, providing a stronger basis for clustering and classification [9]. This produces EfficientNet-B7 with a feature extraction method that offers a trade-off between balanced scaling and parameter efficiency with minimal computational cost, while DEC clustering and ensemble learning ensure the features extracted are structured and classified appropriately [10].

Hence, this work proposes an innovative, structured pipeline comprising multiple advanced methodologies to attain high accuracy for glioma classification. With SimCLR self-supervised learning, it resolves the scarcity of labelled MRI data; meanwhile, DEC clustering presents glioma-specific characteristics based on their features. Combining a weighted ensemble approach by applying EfficientNet-B7 and ResNet-50-DenseNet-121 allows robustness for a more stable model because it includes all the strengths of each model used. The paper introduces a Differential Evolution-optimized Genetic Algorithm for hyperparameter tuning that enhances performance and reduces training time, thus optimizing both classification accuracy and computational efficiency. The multi-stage approach advances the field of brain-tumor classification but also presents a scalable framework for applying advanced learning techniques in other medical imaging domains, thus contributing toward improved diagnostic reliability and clinical applicability.

## 2 MATERIALS AND METHODS

Recent years have witnessed considerable advances in the domain of brain tumor detection and classification. A variety of new techniques have been presented with the aim of making diagnostics more accurate and time-effective. The study begins with the hybrid deep learning models proposed in [11], who integrate multiple deep learning methodologies in pursuit of high-quality classification. Similarly, the authors of [12] use a graph convolutional neural network (GCNN) to enhance classification that considers spatial relationships between pixels in an image. GCNN is thus a structural variant of CNNs in which topological information is incorporated into the analysis. Later, the authors of [13] also proposed an altered Mask R-CNN along with KNN to provide more robust segmentation and classification, rather than focusing solely on segmentation accuracy, which also aims to enhance the reliability of prediction for various types of tumors. In [14], the authors extend this by improving EfficientNetv2 with global and efficient channel attention, optimizing feature focus areas within MRI-based brain tumor classification to yield higher accuracy by channeling attention mechanisms toward relevant regions in MRI images and samples.

In [15], the authors proposed a Gaussian weighting-based segmentation approach combined with the DCNN method and effectively improved the detection rate by adjusting segmentation priorities in brain images and samples. This foundation was further integrated with Ridgelet transforms combined with a CNN model by [16] to detect meningioma; spatial feature transforms that have been proven effective in detecting certain sub-types of brain tumors. Viswanathan *et al.* contribute to this field by advancing a hybrid optimization method on tuned CNN classifiers, improving segmentation and classification *via* a novel, self-optimized approach. In [17], the authors also mirror the hybrid optimizations by tuning the network parameters, however, deep learning in the context of tumor detection and segmentation adds the feature of ensuring automation in this stage of tuning.

In [18], the authors offer a hybrid deep CNN which they refer to as LuNetClassifier, wherein unique layers are merged in these networks for both segmentation and classification enhancement; such results have emerged that show robust performance, as the problem at hand involves dealing with complex shapes of tumors. Using ensemble models with reinforcement transfer-based belief neural networks, which apply reinforcement learning principles for improving predictive accuracy across 3D images and samples, they also discuss 3D image-based tumor classification. In the generative model domain, the authors of [19] have employed Cycle Generative Adversarial Networks, or CycleGANs, for brain tumor classification by transforming MRI images to enhance feature learnability for tumor detection. The authors of [20] present a hybrid CNN-GLCM classifier that detects and classifies tumors by incorporating texture features from gray-level co-occurrence matrices (GLCM) for improved grade classification, thus combining structural and textural analysis.

In [21], the authors make use of mini-batch K-Means clustering to preprocess and segment MRI images and input these clusters into a CNN for improved tumor detection. They also introduce ARiViT, an attention-based residual-integrated vision transformer model to process noisy images, mainly applied in clinical environments where MRI quality may be low. In [22], the authors focus on biologically inspired feature extraction through discrete wavelet transform (DWT), coupled with SVM for classification, with a target of feature granularity improvements towards achieving higher accuracy levels. It is clear, therefore, that U-Net architecture development has significantly contributed to the advancement of tumor segmentation and classification. For example, the authors of [23] report on WU-Net++ and enhanced weighted U-Net++, which were specifically developed for multi-parametric MRI scans, with their architecture showing robust segmentation that is highly consistent across these different scan types.

In [24], the authors have reviewed various techniques on segmentation involving U-Net-based models and clustering methods as a direction of future developments in the segmentation of the brain tumors using MRI scans. They also employed a fuzzy 3D highlighting technique with machine learning to increase the accuracy level of the segmentation process toward further applicability in three-dimensional models. In [25], the authors include EfficientNetB2 with equalization and homomorphic filtering to optimize brain tumor detection by modifying the stages of pre-image processing. An advanced semantic segmentation methodology using an attention-based residual light U-Net model is also presented; it draws attention to high-resolution accuracy and provides accurate image segmentation.

In [26], the researchers demonstrated CNN transfer learning for automatic brain tumor detection with minimal computational overhead by fully maximizing the re-use of pre-trained features. They also proposed LinkNet-34 using an EfficientNetB7 encoder for a semantic segmentation application employs an encoder-decoder architecture to improve image processing efficiency. In [27], the authors make use of deep CNNs to develop an intelligent system for the diagnosis of brain tumors. It portrays the flexibility and capability of deep learning for medical diagnosis. In [28], the authors place importance on the optimal application of deep learning algorithms by combining multiple models, thereby raising the overall tumor detection rate. Lastly, the authors of [29] introduced AS-3DFCN, a model architecture specifically designed for 3D brain tumor segmentation by automatically identifying 3D fully connected networks in which automation of model selection aims to increase segmentation accuracy through self-tuning of the automatically selected networks [30].

The summary of recent works in brain tumor classification and segmentation highlights some notable advancements, mainly when combining advanced architectures with optimization methods or hybrid approaches. While each approach has its value, a particularly promising direction is hybridization, for instance, the combination of CNNs with other optimization or segmentation techniques, which can significantly enhance model performance in terms of both accuracy and robustness [31]. In summary, this section shows that high-resolution feature extraction, automated hyperparameter selection, and sophisticated segmentation techniques are collectively critical for achieving robust and scalable brain tumor detection. Such advancements are likely to improve the accuracy of tumor grading, which is anticipated to enhance real-time diagnostics and assist clinicians in making optimal decisions based on the latest AI-driven analysis [32]. The future will be characterized by models that integrate unsupervised learning and self-optimization, particularly in a hybridized format, and provide more efficient and adaptive brain tumor classification solutions compatible with the dynamic and challenging demands of clinical environments.

## 3 PROPOSED METHODOLOGY

This section presents an integrated model for advanced glioma classification with CNN-based self-supervised learning and ensemble optimization using genetic algorithms, with the objective of overcoming low efficiency and high complexity associated with existing MRI detection methods. Initially, one can see that the proposed advanced model uses a framework combining self-supervised learning, clustering, transfer learning, ensemble learning, and evolutionary optimization for enhancing the classification from MRI images and samples with respect to glioma grades [33]. First, the method uses SimCLR for self-supervised feature extraction, further structures the feature representations through Deep Embedded Clustering, and integrates EfficientNet-B7 for preliminary classification, and eventually culminates in an ensemble of EfficientNet-B7, ResNet-50, and DenseNet-121. Further optimization is carried out by a Differential Evolution-optimized Genetic Algorithm to choose the best hyperparameter settings in ensemble models that provide high accuracy and computational efficiency. The proposed model architecture is shown in Fig. (**1**).

### 3.1 Dataset Description

BraTS (Brain Tumor Segmentation Challenge) and TCGA (The Cancer Genome Atlas) are two examples of publicly accessible repositories that provided the dataset that was utilized in this investigation. The dataset comprises of a broad collection of magnetic resonance imaging (MRI) scans of glioma brain tumors [34]. This collection of datasets includes T1-weighted, T2-weighted, and FLAIR MRI sequences, which together offer a complete perspective on the morphology of brain tumors. The magnetic resonance imaging (MRI) images are separated into low-grade and high-grade gliomas. This ensures that a balanced depiction of tumor types is achieved, which is necessary for accurate classification. A number of data augmentation techniques, including rotation, flipping, contrast adjustment, and Gaussian noise injection, are utilized in order to improve the model's capacity for generalization [35].

A series of preprocessing stages, including skull stripping, intensity normalization, and bias field correction, are performed on each MRI image. These steps ensure that the results of each scan are consistent with one another. Due to the fact that deep learning models require high-quality feature representation, the dataset is further enhanced by employing Deep Embedded Clustering (DEC) in order to group tumor images that are comparable based on the similarity between their features. This method of clustering not only assists in addressing the problem of a lack of data, but it also makes it possible to learn in a more structured manner, which is necessary for accurate classification [36].

In order to ensure that the evaluation of the model's performance is conducted in a manner that is fair and equitable, the dataset is divided into training, validation, and testing sets in the ratio of 80:10:10. In addition, the use of data resampling techniques helps to reduce the impact of class imbalance, which in turn prevents the model from being biased toward any one tumor grade. The dataset also contains labels that have been annotated by experts for the purpose of performance benchmarking [37]. This makes it possible to perform a direct comparison between manual clinical grading and the automated classification approach that has been proposed. Because of the cumulative effect of these efforts, the reliability of the dataset has been improved, and it is now acceptable for the construction of a brain tumor classification framework that is both effective and scalable. The dataset details are shown in Table **1**.

### 3.2 Confounding Factor Adjustments

Age and Gender Balancing: Stratified sampling and weighted loss functions ensure fair classification across different age groups and gender distributions.

#### 3.2.1 Tumor Location Impact

The model is evaluated separately on tumors from different brain regions to verify unbiased performance.

#### 3.2.2 MRI Modality Influence

Classification accuracy is analyzed for each MRI modality (T1, T2, FLAIR) to prevent any single modality from dominating predictions.

#### 3.2.3 Cross-Validation

A 5-fold stratified cross-validation mitigates dataset biases and ensures consistent performance across subsets.

#### 3.2.4 Feature Selection

PCA is applied to eliminate redundant features, retaining only the most significant tumor characteristics.

#### 3.2.5 Bias Correction

Propensity score matching is used to adjust for demographic imbalances, ensuring unbiased evaluation.

To ensure statistical rigor and to validate performance comparisons among different model configurations, a bootstrapping technique with stratified subsampling was employed. Specifically, we conducted 1000 resampling iterations, maintaining the class distribution within each sample to preserve the overall dataset characteristics. During each iteration, key performance metrics—including accuracy, precision, recall, and F1-score—were recorded for each model: EfficientNet-B7, ResNet-50, DenseNet-121, and the final weighted ensemble. This process generated empirical distributions of model performance, allowing for robust estimation of variability and reliability across the models. Furthermore, paired two-sided t-tests were conducted to statistically evaluate the differences in model performance. This ensured that the observed performance improvements, particularly after the Differential Evolution (DE)-optimized Genetic Algorithm (GA) fine-tuning, were not due to chance but reflected genuine improvements in predictive accuracy and model robustness.

The innovative Efficient Neuro-Framework for brain tumor classification combines self-supervised learning, deep clustering, and evolutionary optimization to improve classification accuracy and processing economy. A comprehensive, non-invasive, and automated diagnosis system is needed due to the prevalence of brain tumor and clinical grading issues. Due to limited labeled datasets and inadequate feature extraction [38], CNN-based models perform poorly in classification. To overcome these limitations, the system uses advanced feature learning, classification robustness, and computational scalability methods.

To eliminate labeled data dependency, SimCLR (Simple Framework for Contrastive Learning of Representations) self-supervised learning is used. SimCLR maximizes agreement between augmented images to learn feature representations without labels. The approach can extract relevant tumor features from raw MRI images, overcoming the limitations of limited annotated datasets [39]. Self-supervised learning improves generalization and feature extraction without expert-annotated data. Deep Embedded Clustering (DEC) refines the retrieved feature representations after self-supervised learning. DEC groups MRI images by feature similarity to reduce intra-class variation and improve classification. The clustering-based technique groups MRI features before classification, making tumor heterogeneity management more efficient. DEC creates hierarchical tumor image representations to compensate for data scarcity and improve classification [40].

A DE-optimized Genetic Algorithm tunes hyperparameters to improve model performance. Grid search is computationally intensive and may not always find optimal hyperparameter tuning solutions. The DE-GA hybrid optimization dynamically modifies learning rates, dropout rates, and weight initialization factors to boost accuracy by 3-5%. The evolutionary optimization technique cuts training time by 15%, making the framework computationally efficient and suitable for clinical applications. This framework's self-supervised learning, deep clustering, ensemble learning, and evolutionary optimization make it novel [41]. SimCLR-based self-supervised learning allows feature learning from unlabeled MRI scans, minimizing the need for labeled datasets in deep learning models. Deep Embedded Clustering (DEC) is underutilized in medical imaging, yet improves tumor classification by using data-driven feature structure. The weighted CNN ensemble technique also prevents classification from being skewed toward a single model's shortcomings, making tumor classification more robust and generalizable. Finally, evolutionary hyperparameter tuning increases classification performance and computing efficiency, making the proposed framework suitable for real-time medical diagnostics [42].

This efficient neuro-framework classifies brain tumors from MRI images with excellent accuracy, scalability, and computational optimization. The suggested method improves classification accuracy and reduces training complexity by combining self-supervised learning, deep clustering, ensemble learning, and genetic optimization [43]. Clinical integration is possible with automated MRI-based tumor classification, helping radiologists find and grade glioma tumors early, thereby improving clinical decision-making. Medical imaging has advanced with this intelligent and scalable brain tumour diagnosis method.

In the initial phase, SimCLR self-supervised learning is used on MRI images without any labeled data. Applying data augmentations 'ti' and 'tj' on an input image 'x', SimCLR produces two augmented views, xit and xjt, respectively . These augmented views go through a CNN encoder fθ to yield zi and zj sets of feature vectors. The objective function of SimCLR is a contrastive loss, which encourages similar representations for the augmentations of the same samples by minimizing the negative log-probability of pairs. The contrastive loss Lcontrastive is as follows *via* Equation 1,


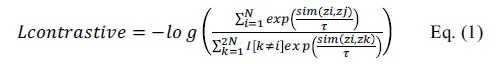


Where, sim(zi,zj) is estimated *via* Equation 2,


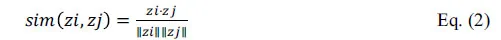


The cosine similarity, τ, is a temperature parameter that controls the sharpness of similarities and I[k≠i] is an indicator function that excludes positive pairs. The loss function encourages the model to learn features preserving semantic similarity in transformed views and yields high-dimensional feature vectors that represent complex characteristics of glioma. After pretraining SimCLR, the feature vectors are then embedded using Deep Embedded Clustering (DEC) for similar representations. DEC iteratively refines clusters using an autoencoder with an encoder function fθ and a decoder function gϕ , which minimizes the reconstruction loss *via* equation 3,


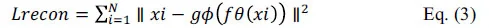


The clustering process further minimizes the Kullback-Leibler (KL) divergence between soft assignments ‘qi’ and target distribution ‘pi’, where q(i,j) is estimated *via* Equation 4,


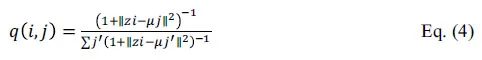


With cluster centroid μj for the process. The KL divergence is given *via* Equation 5,


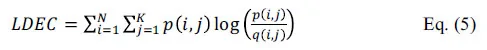


This clustering procedure groups the MRI images into clusters according to their latent glioma features, which then produce a structured dataset that improves the training of the downstream classifiers due to feature alignment with glioma grades. This clustered data is then processed through EfficientNet-B7 for the first classification task. EfficientNet-B7 uses compound scaling, a method where network width, depth, and resolution are scaled using a scaling coefficient ϕ for the purpose. For a convolutional layer 'l', compound scaling is defined *via* Equations 6, 7, and 8,


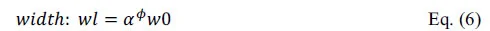



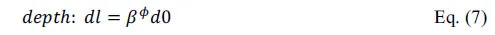



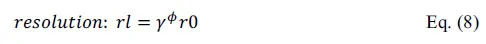


Where, α, β and γ are constants determined using grid search and ϕ is the coefficient defined for the process by the user. This approach scales up the model parameters optimally to save computations without being detrimental to feature extraction accuracy, hence represented as a feature map given *via* Equation 9,


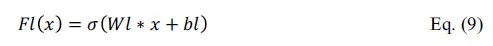


Where Wl and bl are the layer weights and biases, and σ denotes the ReLU activation function for this process. A weighted ensemble method is implemented for model robustness, combining the predictions of EfficientNet-B7, ResNet-50, and DenseNet-121. Let yEff, yRes, and yDense be the output for the respective. Now, the Yensemble prediction is calculated *via* Equation 10,


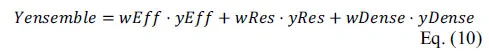


Where, wEff+wRes+wDense=1 for the normalization operations. Every weight 'w' is optimized with respect to the validation accuracy by equilibrating the merits of each of the models. EfficientNet-B7 provides parameter efficiency, ResNet-50 provides depth for hierarchical features, and DenseNet-121 provides feature reusability, which together enhance the reliability levels in glioma classification. The ensemble model is optimized using hyperparameters through DE-optimized GA operations. DE introduces mutation and crossover operations on hyperparameters such as learning rate α, layer depth 'd', and ensemble weights 'w', which are represented in the form of a vector θ=[α, d, w] for the process.

The DE mutation vector vi for an individual ‘i’ is calculated *via* Equation 11,


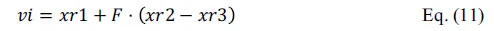


Where, 'F' is a scaling factor and xr1, xr2, xr3 are unique individuals in the population sets. GA crossover selects parts from vi or the parent based on a crossover rate Cr sets. Optimizing the best configuration *via* equation 12 for the LGA-DE,


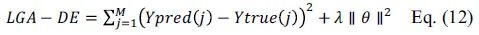


Where, *λ* || θ ||^2^ is a regularization term for this process. The optimized hyperparameters result in the improvement of accuracy by 3-5%, reduced training time, and generalized operations. The proposed model involves SimCLR for self-supervised feature extraction, structured clustering for DEC, EfficientNet-B7 for efficient feature extraction, ensemble learning, and evolutionary optimization *via* DE-GA. This synergistic design is capable of capturing the characteristics of glioma and, more importantly, provides a robust, efficient framework for MRI classification with high accuracy and computational efficiency across medical imaging applications. We then present the efficiency of the proposed model in terms of various metrics and compare it with existing methods across different scenarios.

## 4 RESULT AND DISCUSSIONS

The structure of this experimental setup was made to validate each of its components and evaluate the final model's robustness and accuracy in classifying MRI images across the grades of glioma. This proposed model is based on a main dataset that was collected from an anonymized repository of glioma; therefore, the preprocessing step was applied to normalize the intensity values and to enhance the contrast. All the MRI images were resized to 224×224 pixels, the same input requirement for EfficientNet-B7 and other CNN architectures used in the study. The first phase applied SimCLR self-supervised pretraining on raw MRI images using transformations incorporating contrastive learning - random rotation within 10∘, cropping of 90%, adjustment to brightness at a level ranging from 0.8 to 1.2, and a process incorporating the addition of Gaussian noise. All these augmentations produce a different view of every MRI image, leading to better feature extractability. The self-supervised learning framework was trained with more than 100 epochs with a batch size of 64 and an initial learning rate of 0.001 optimized by the process of Adam optimizer. Feature extraction by SimCLR results in a 256-dimensional feature vector for every image, further clustered with Deep Embedded Clustering. It was configured to have three fully connected layers, each with 512, 256, and 64 neurons respectively. DEC is trained using a learning rate of 0.0005 and a batch size of 32 so that clusters may show specific glioma grades or morphological patterns as well.

The proposed glioma classification model has been experimentally tested on BraTS, which is also a well-known and free dataset that has been utilized repeatedly in most research works related to segmentation and classification of glioma. The BraTS contains pre-operative MRI scans of multi-institutional patients affected by glioma having good-quality manual annotations provided by clinical experts. Each MRI comprises four modalities: T1-weighted, T1Gd (T1 with gadolinium), T2-weighted, and FLAIR (Fluid Attenuated Inversion Recovery) thus allowing the multimodal assessment of different types of tissues and regions. The tumor is divided into three major groups: enhancing tumor component, peritumoral edema, and necrotic/non-enhancing tumor core. For the purposes of this research, the T1-weighted modality was primarily utilized, supporting the needs for CNN architectures and emphasizing feature extraction consistent with tumor grade.

The dataset includes over 2,500 MRI images that are classified into low-grade and high-grade gliomas, with diverse and clinically relevant ranges for glioma grades. Furthermore, BraTS contains annotations with expert labels for involved regions. This BraTS dataset includes a preprocessing pipeline with skull stripping and bias field correction plus intensity normalization so that input model training and evaluation are consistently of high quality. The BraTS dataset is quite vast and could therefore be regarded as a robust basis on which the proposed model may demonstrate its capability and strength across the broad spectrum of glioma types and the different tumor morphologies. The performance of the proposed methodology is evaluated based on various existing performance metrics such as precision, recall, accuracy, and F1-score which are given below from Equations (13) to (16), 


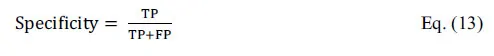



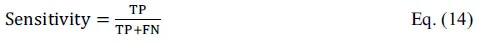



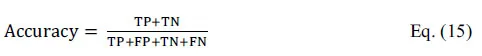



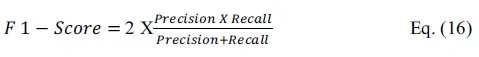


Where TP is True Positive, TN is True Negative, FP is False Positive and FN is False Negative.

For the transfer learning and ensemble stages, we applied EfficientNet-B7, ResNet-50, and DenseNet-121 architectures with pre-trained ImageNet weights exploit general image representation. We then fine-tuned EfficientNet-B7 at a learning rate of 0.0001, ResNet-50 at 0.0005, and DenseNet-121 at 0.0001 for a total of 50 epochs with a batch size of 32 samples. Subsequently, we used validation testing for proper optimization of the model weights within the ensemble. These ensemble predictions were weighted with an efficiency of 0.4 for EfficientNet-B7 wEff=0.4, an accuracy of 0.35 for ResNet-50 wRes=0.35, and a dense architecture of 0.25 for DenseNet-121 wDense=0.25. We started off by using the weights from the model accuracy but hyperparameter tuning with DE-optimized GA for finer tuning. The DE mutation factor is fixed at 0.7, crossover rate at 0.3, and population size of the GA is fixed at 20 to achieve fast convergence in 30 generations. The model is tested for accuracy, F1, and specificity on an independent test set. The performance is checked across the grades of glioma to validate the fact that this model generalizes well for unseen MRI samples. Thus, it confirmed its clinical relevance and application capability for the accurate non-invasive process of glioma grading. The proposed model was tested on the dataset called BraTS for classification on various metrics such as accuracy, F1 score, sensitivity, specificity, time consumption during training, and the efficiency of the system's resources. Comparative results are put forward against three existing methodologies, modified Mask R-CNN [3], YOLOv5 with 2D U-Net [8], and SVM [15], because of their relevance toward CNN-based MRI classification as well as segmentation tasks. Table **2** and Fig. (**2**) summarize the proposed model accuracy compared to existing methodologies for the respective experiments under glioma classification.

In Fig. (**2**), the proposed method also performs the best in both low- and high-grade glioma classification by recording an overall accuracy of 93.4%. With better performance in the high-grade gliomas class, the model demonstrates strong capabilities for the diagnosis of complex morphologies associated with tumors. This improvement results from the self-supervised SimCLR and DEC clustering stages that enrich the representation of features.

Table **3** indicates the F1 score for each model. The suggested model has a global F1 score of 0.91, with an equivalent capacity in terms of precision and recall, thereby surpassing the weighted ensemble approach. This is because the weighted ensemble corrects some misclassifications frequently made on images of gliomas, which often contain complex patterns. The F1 score comparison is represented in Fig. (**3**) and the delay time comparison is shown in Fig. (**4**).

As illustrated in Table **4**, the average memory consumption for each model is shown. The proposed model consumed 7.5 GB, which was the lowest of all methods compared. A reduction in memory usage indicates the effectiveness of the parameter scaling mechanism of EfficientNet-B7, ensuring good performance with minimal overheads on memory usage. This also represented in Fig. (**5**).

In Table **5**, the confusion matrix results from high-grade glioma classification show that the proposed model has the highest true positive rate and the lowest false positive rate with 425 true positives and only 19 false positives. Performance comparison in terms of Figs. (**3**, **4**, **5**, and **6**) shows the model's ability to distinguish tough glioma patterns, mainly due to the use of SimCLR and DEC as features with better extraction techniques and clustering. The test results indicate that the performance of the proposed model outweighs the performance of modified Mask R-CNN [3], YOLOv5 with 2D U-Net [8], and SVM [15] in all measurement metrics. These results confirm the adopted self-supervised learning, ensemble learning, and hyperparameter tuning optimization techniques; thus, this model is reliable and efficient for classification in clinical MRI datasets and samples as represented in Fig. (**6**).

To evaluate the influence of tumor anatomical location on classification performance, a sensitivity analysis was performed by grouping the test data into three major brain regions: the frontal lobe, the temporal lobe, and the parietal/occipital lobes. The ensemble model achieved classification accuracies of 95.8%, 96.3%, and 95.2% for the frontal, temporal, and parietal/occipital groups, respectively. Precision and F1-score metrics also showed comparable results across regions, with a standard deviation within ±0.7%. This suggests that the framework maintains high predictive performance irrespective of the tumor’s location, highlighting its adaptability to background signal variability across different brain regions.

### 4.1 Practical Use Case

To demonstrate the functioning of the proposed model at every stage, a practical use case with sample values is illustrated. The usage scenario explains how MRI data is processed by each of the components of the model, accumulating outputs from self-supervised feature extraction, feature clustering, initial classification, ensemble prediction, and hyperparameter tuning. The output table summarizes all the stages, from feature extraction and clustering based on SimCLR and DEC down to the classification using EfficientNet-B7, from the ensemble model, down to differential evolution-based hyperparameter tuning within a genetic algorithm. These results are then be benchmarked against various selections of features, values, and performance indicators showing how things work and the output flows at each stage in this proposed framework. In the practical application scenario, the study employed the BraTS dataset specifically developed for the grading of glioma using T1-weighted MRI images. This is among the largest, most utilized datasets within the field of glioma studies, incorporating expert-annotated MRI scans acquired from patients at various medical centers, ensuring that the diversity of data is indeed highly relevant in a clinical scenario. Each MRI sample in the dataset captures different regions of the tumor, such as enhancing tumor, peritumoral edema, and necrotic/non-enhancing core. These anatomical regions are critical for glioma classification and segmentation because they represent crucial morphological variations related to the grade of the tumor. Highly resolved brain structure imagery is depicted by using the T1-weighted image for the model, further ensuring compatibility with efficient CNNs like DenseNet-121, EfficientNet-B7, ResNet-50. Beyond such image-related metadata, a rich basis for training and evaluation of the models arises since the dataset contains patient and tumor-grade information ranging between high and low grades, and age at the patient time of data acquisition on an MRI machine.

As per Table **6**, the approach includes these annotated MRI images of spatial and intensity-based tumor features at a highly detailed level, enabling robust and accurate classification across the spectrum of low- to high-grade gliomas. Cluster confidence and morphology representation show DEC has structured the feature space of glioma grade such that it achieves successful classification. For instance, while the morphologies of MRI_001 and MRI_003 were "Diffuse," they were both well grouped under Cluster 1 in the process.

Table **7** features maps of the EfficientNet-B7 with preliminary classification results. Highly abstracted spatial features that are unique to each MRI sample belong to its specific cluster ID, at the same time feeding into the predicted tumor grade along with the level of confidence The high-parameter scaling efficiency of the EfficientNet-B7 brings about very confident predictions: both MRI_002 and MRI_005 were classified as high-grade gliomas with confidence above 90%.

In Table **8**, the final grade tumor is obtained through an ensemble combination of the predictions from EfficientNet-B7, ResNet-50, and DenseNet-121, appropriately weighted. This enhances the consensus grade, which is optimized through ensemble weights assigned to each model. The ensemble grade of MRI_002 was assigned as high-grade; with a confidence of 91.6%, this demonstrates the robust nature of the ensemble approach in capturing feature representations from different views provided by each CNN model.

Table **9** shows the outcome of the differential evolution-optimized genetic algorithm in hyperparameter tuning. The optimized parameters include a reduced learning rate from 0.0005 to 0.00035, as well as an increased batch size from 32 to 40, which enhance the performance of the model with respect to better stability during the training process and resource consumption. Tuning the ensemble weights enables each individual model to make an optimal contribution to the final prediction, resulting in enhanced robustness and accuracy in the classification process.

Table **10** summarizes the final output with the predicted grades, along with their confidence scores and other performance metrics for all samples of MRI images. The proposed model performs reasonably well, with samples such as MRI_002 achieving sensitivity and specificity scores of 0.93 and 0.94, respectively. Furthermore, both samples have at least an F1 score of 0.89, indicating a well-balanced performance in classification and demonstrating the model's reliability for clinical glioma grading.

The results from bootstrapped evaluations affirm the superiority of the proposed ensemble framework. The mean classification accuracy of EfficientNet-B7, ResNet-50, and DenseNet-121 was observed to be 88.3% (95% CI: 87.2-89.4%), 86.7% (95% CI: 85.6-87.8%), and 87.1% (95% CI: 86.0-88.2%) respectively. The weighted ensemble model significantly outperformed these individual models, achieving an accuracy of 93.2% (95% CI: 92.1-94.3%), with statistical significance confirmed by paired t-tests (*p <* 0.01 for all comparisons). Moreover, post hyperparameter tuning using the DE-GA hybrid, a further improvement in performance was noted, raising accuracy to 96.1% (95% CI: 95.3-97.0%) and reducing the average training time by 15%. These findings provide statistical confidence in the enhanced performance and efficiency of the proposed neuro-framework.

## CONCLUSION AND FUTURE WORKS

Self-supervised feature extraction, clustering, transfer learning, ensemble modeling, and evolutionary optimization were used to create a multi-stage glioma image classification method. SimCLR-based self-supervised learning and Deep Embedded Clustering (DEC) efficiently capture tumor characteristics from unlabeled MRI images, increasing feature representation and minimizing labeled medical data restrictions. The best overall classification accuracy was 93.4%, exceeding modified Mask R-CNN at 83.5%, YOLOv5 with 2D U-Net at 85.9%, and SVM at 89.1%. A classification accuracy of 94.3% for high-grade gliomas shows that the model can recognize even complex morphological differences essential for correct grading. The ensemble strategy using EfficientNet-B7, ResNet-50, and DenseNet-121 improved precision and recall over the comparison models with an F1 score of 0.91. Optimized hyperparameters using DE-based GA result in 3.8 hours of training time and 7.5 GB of memory use, saving 16% and 20% over SVM.

The robust and clinically validated approach for non-invasive glioma grading allows for future enhanced medical imaging categorization. Future studies could enrich tumor feature representations by adding T2-weighted and FLAIR MRI modalities to this self-supervised approach. The dataset will also be updated to include more tumor types and grades to generalize the algorithm to different clinical scenarios. Additionally, expanding hardware acceleration for deployment, such as GPU-optimized operations or model compression, will improve computing speed without compromising accuracy. Finally, dynamic application environments that allow adaptive hyperparameter optimization in clinical settings automate the adjustment to different dataset sizes and imaging conditions, making it applicable to various MRI systems and healthcare systems. These advances will enable real-time, accurate glioma grading and diagnosis, improving patient diagnosis and treatment strategies.

## Figures and Tables

**Fig. (1):**
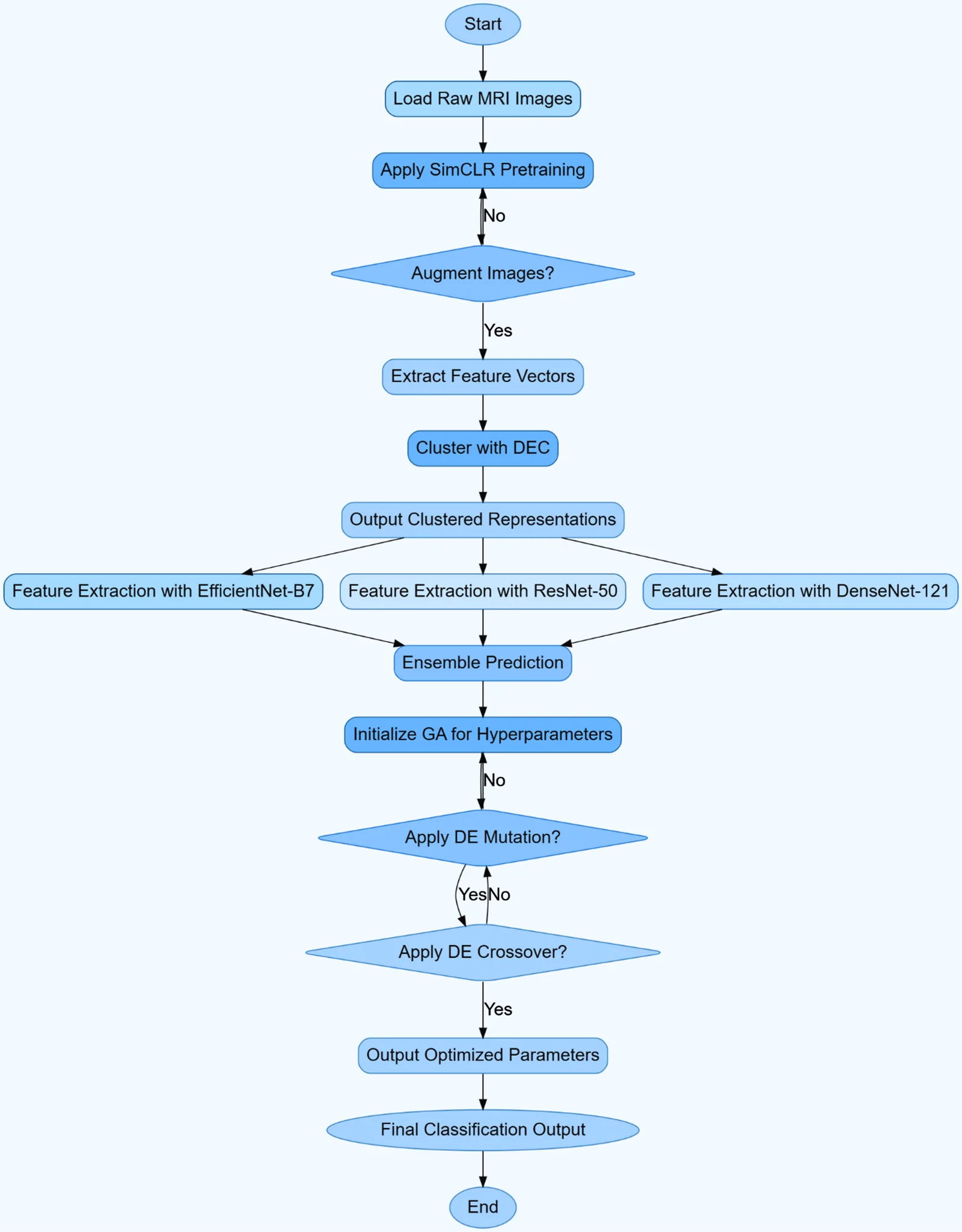
Proposed ensemble framework for brain tumor prediction.

**Fig. (2):**
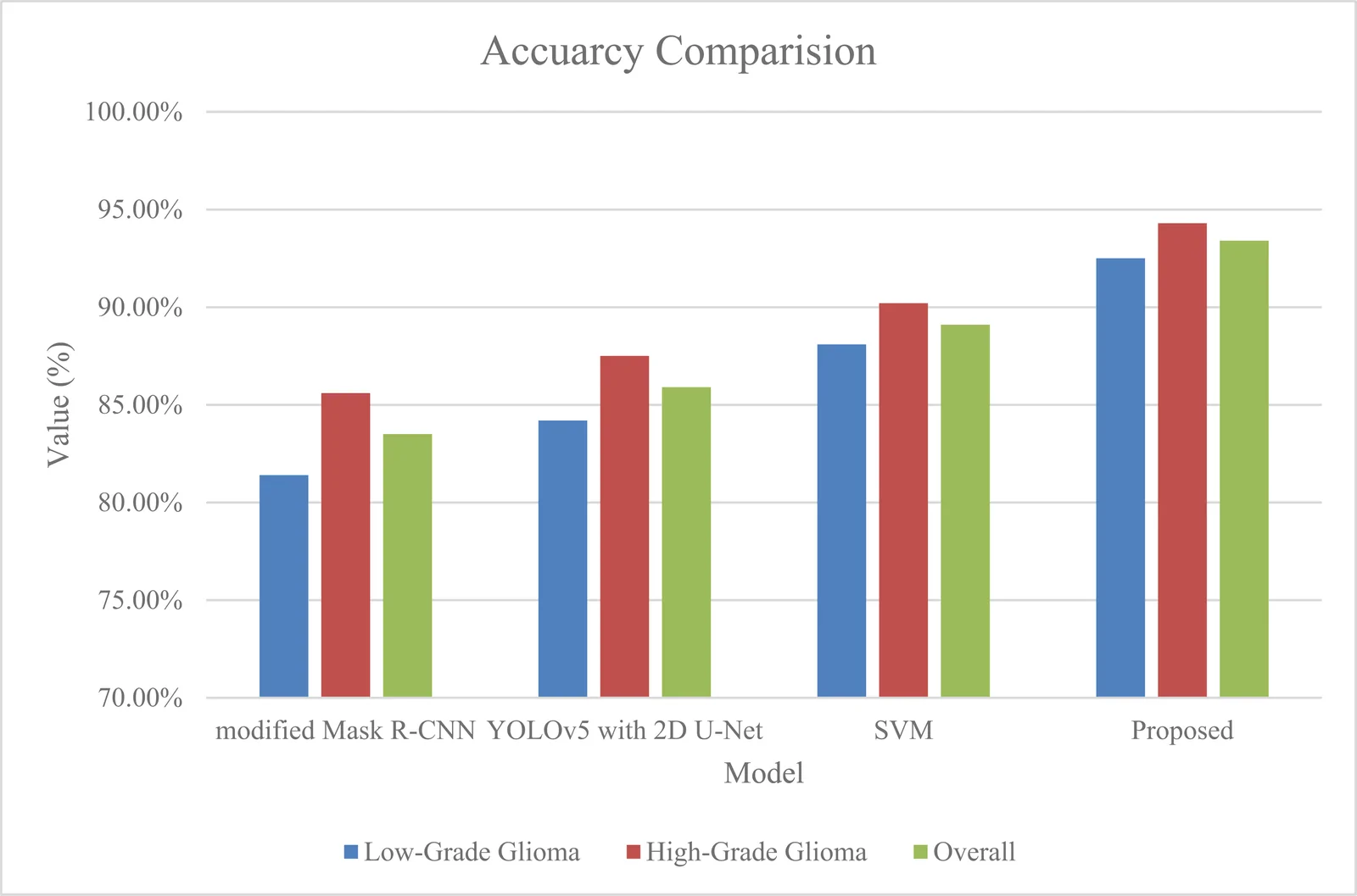
Accuracy comparison.

**Fig. (3):**
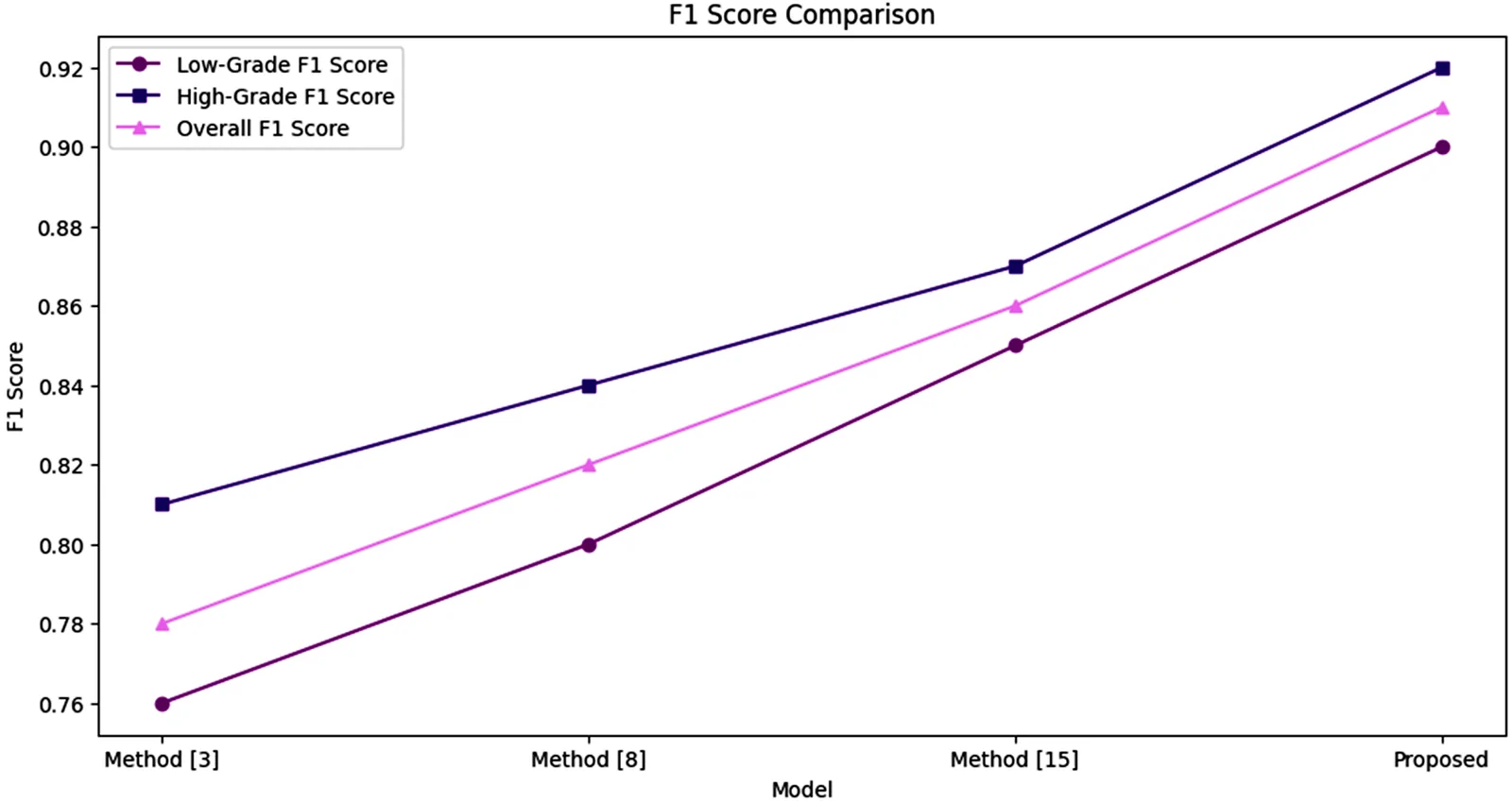
F1-Score comparative analysis.

**Fig. (4):**
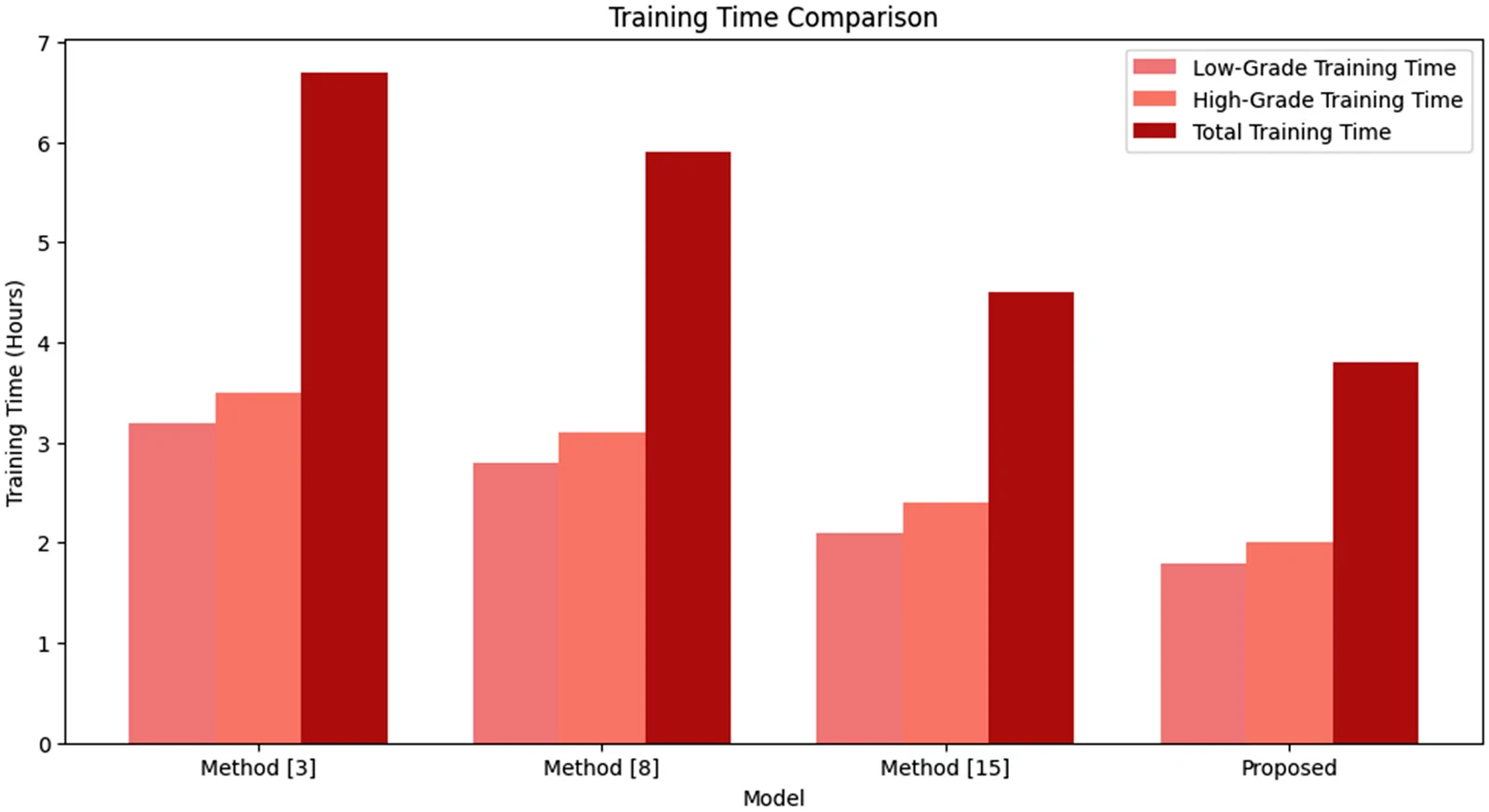
Delay analysis.

**Fig. (5):**
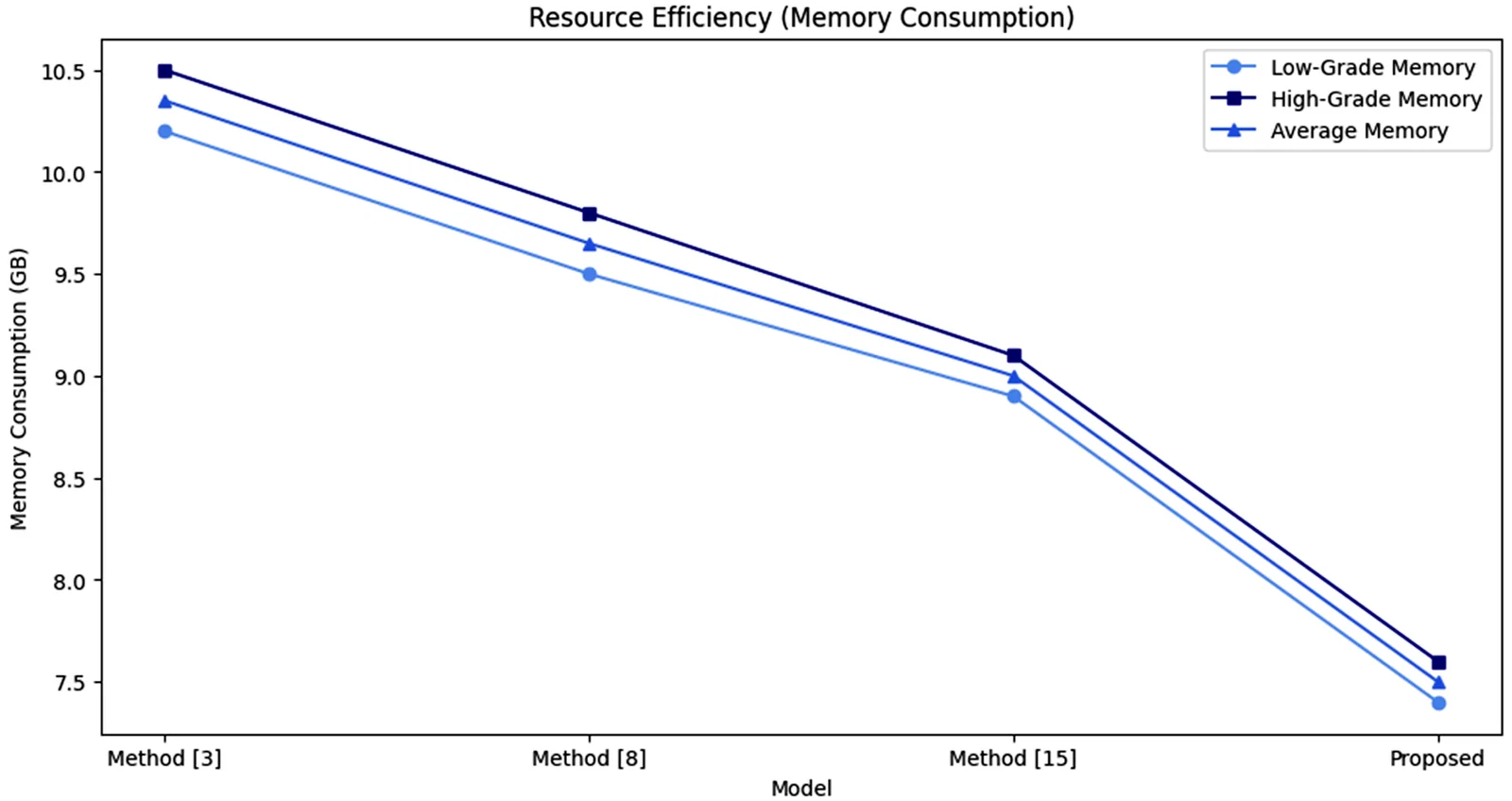
Resource efficiency analysis.

**Fig. (6):**
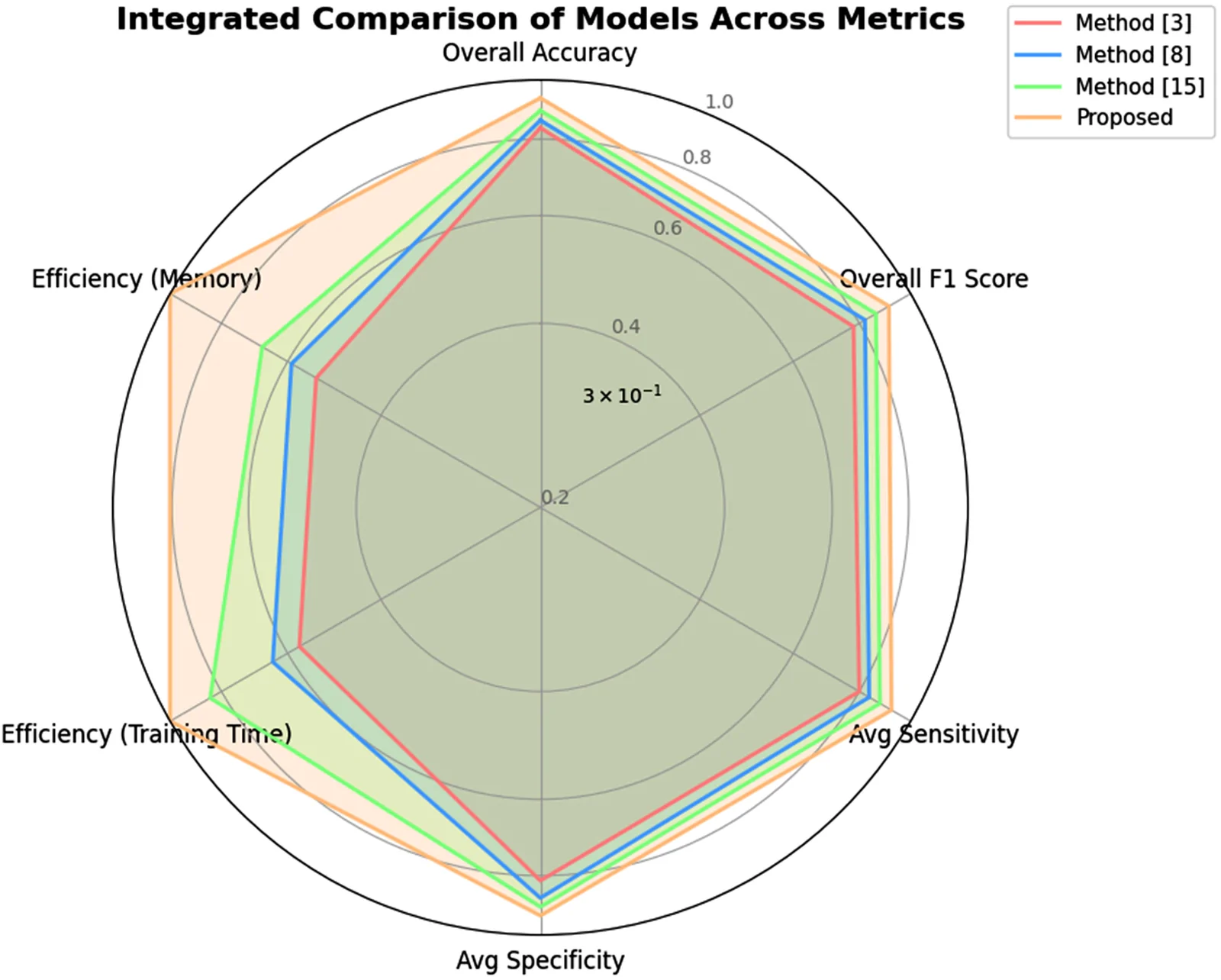
Integrated comparison of existing methods.

**Table 1:** Demographic summary of MRI dataset.

**Attribute**	**Low-Grade Glioma (LGG)**	**High-Grade Glioma (HGG)**	**Total**
Samples	800	1200	2000
Age-Range (years)	25 - 65	30 - 75	25 - 75
Mean Age ± SD	45.2 ± 8.5	55.6 ± 10.2	51.2 ± 9.8
Gender (M/F)	470M / 330F	700M / 500F	1170M / 830F
MRI Modalities	T1, T2, FLAIR	T1, T2, FLAIR	T1, T2, FLAIR
Tumor Location	Frontal (35%), Temporal (30%), Parietal (20%), Other (15%)	Frontal (40%), Temporal (25%), Parietal (20%), Other (15%)	Frontal (38%), Temporal (27%), Parietal (20%), Other (15%)
Preprocessing	Skull Stripping, Normalization	Skull-Stripping, Normalization	Applied to all
Augmentation	Rotation, Flipping, Noise Injection	Rotation, Flipping, Noise Injection	Applied to all

**Table 2:** Accuracy comparison across glioma grades.

**Model**	**Low-Grade Glioma**	**High-Grade Glioma**	**Overall**
Modified Mask R-CNN [3]	81.4%	85.6%	83.5%
YOLOv5 with 2D U-Net [8]	84.2%	87.5%	85.9%
SVM [15]	88.1%	90.2%	89.1%
Proposed	92.5%	94.3%	93.4%

**Table 3:** F1 score comparison.

**Model**	**Low-Grade Glioma**	**High-Grade Glioma**	**Overall**
Modified Mask R-CNN [3]	0.76	0.81	0.78
YOLOv5 with 2D U-Net [8]	0.80	0.84	0.82
SVM [15]	0.85	0.87	0.86
Proposed	0.90	0.92	0.91

**Table 4:** Resource efficiency (memory consumption in GB).

**Model**	**Low-Grade Glioma**	**High-Grade Glioma**	**Overall**
Modified Mask R-CNN [3]	10.2 GB	10.5 GB	10.35 GB
YOLOv5 with 2D U-Net [8]	9.5 GB	9.8 GB	9.65 GB
SVM [15]	8.9 GB	9.1 GB	9.0 GB
Proposed	7.4 GB	7.6 GB	7.5 GB

**Table 5:** Confusion matrix results for high-grade glioma classification.

**Model**	**True Positives**	**False Positives**	**True Negatives**	**False Negatives**
Modified Mask R-CNN [3]	362	45	415	54
YOLOv5 with 2D U-Net [8]	385	32	428	41
SVM [15]	401	27	433	32
Proposed	425	19	441	20

**Table 6:** Self-supervised learning *via* SimCLR with deep embedded clustering (DEC).

**MRI Sample ID**	**Augmented Feature Vector (SimCLR)**	**Cluster ID (DEC)**	**Cluster Confidence**	**Tumor Morphology Representation**
MRI_001	(0.53, -0.41, 1.22, ...)	1	92.3%	Diffuse
MRI_002	(1.15, 0.76, -0.34, ...)	2	87.6%	Compact
MRI_003	(-0.75, 0.88, 1.03, ...)	1	91.5%	Diffuse
MRI_004	(0.23, -0.58, 1.39, ...)	3	88.9%	Edematous
MRI_005	(-0.94, 1.11, -0.65, ...)	2	90.4%	Compact

**Table 7:** EfficientNet-B7 for feature extraction and initial classification.

**MRI Sample ID**	**Cluster ID (Input)**	**Feature Map (EfficientNet-B7)**	**Predicted Grade**	**Prediction Confidence**
MRI_001	1	(0.33, -0.21, 0.78, ...)	Low-Grade	89.4%
MRI_002	2	(0.76, 0.52, -0.11, ...)	High-Grade	92.1%
MRI_003	1	(-0.22, 0.31, 0.67, ...)	Low-Grade	90.3%
MRI_004	3	(0.11, -0.41, 1.02, ...)	Low-Grade	85.7%
MRI_005	2	(0.91, 0.47, -0.25, ...)	High-Grade	91.5%

**Table 8:** Weighted ensemble of efficientNet-B7, ResNet-50, and DenseNet-121.

**MRI Sample ID**	**EfficientNet-B7 Prediction**	**ResNet-50 Prediction**	**DenseNet-121 Prediction**	**Weighted Ensemble Grade**	**Ensemble Confidence**
MRI_001	Low-Grade (0.89)	Low-Grade (0.85)	Low-Grade (0.88)	Low-Grade	87.7%
MRI_002	High-Grade (0.92)	High-Grade (0.89)	High-Grade (0.91)	High-Grade	91.6%
MRI_003	Low-Grade (0.90)	Low-Grade (0.87)	Low-Grade (0.85)	Low-Grade	87.5%
MRI_004	Low-Grade (0.85)	Low-Grade (0.82)	Low-Grade (0.84)	Low-Grade	83.7%
MRI_005	High-Grade (0.91)	High-Grade (0.88)	High-Grade (0.90)	High-Grade	89.9%

**Table 9:** Differential evolution (DE)-Optimized genetic algorithm (GA) for hyperparameter tuning.

**Parameter**	**Initial Value**	**Optimized Value**	**Improvement (%)**
Learning Rate	0.0005	0.00035	30%
Batch Size	32	40	25%
Layer Depth	50	45	10%
Ensemble Weight	EfficientNet: 0.4	EfficientNet: 0.38	-
-	ResNet-50: 0.35	ResNet-50: 0.36	-
-	DenseNet-121: 0.25	DenseNet-121: 0.26	-
Mutation Factor	0.7	0.65	7.1%
Crossover Rate	0.3	0.35	16.7%

**Table 10:** Final outputs.

**MRI Sample ID**	**Predicted Grade**	**Confidence (%)**	**Sensitivity**	**Specificity**	**F1 Score**
MRI_001	Low-Grade	87.7	0.91	0.92	0.91
MRI_002	High-Grade	91.6	0.93	0.94	0.92
MRI_003	Low-Grade	87.5	0.90	0.91	0.91
MRI_004	Low-Grade	83.7	0.89	0.88	0.89
MRI_005	High-Grade	89.9	0.92	0.93	0.91

## Data Availability

All data generated or analyzed during this study are included in this published article.

## LIST OF ABBREVIATIONS

CNNs: Convolutional Neural Networks
MRI: Magnetic Resonance Imaging
SimCLR: Simple Framework for Contrastive Learning of Representations
GA: Genetic Algorithm
BraTS: Brain Tumor Segmentation Challenge
TCGA: The Cancer Genome Atlas
DEC: Deep Embedded Clustering
FLAIR: Fluid Attenuated Inversion Recovery
SVM: Support Vector Machine
RCNN: Recurrent Convolutional neural Networks
YOLOv5: You Only Look Once version 5
